# Practice of Consanguinity and Unusual Cases of Inherited Familial Chromosome Abnormalities: A Case Report

**Published:** 2016

**Authors:** Debarshi Sanyal, Vidya Bhairi, Jayarama S Kadandale

**Affiliations:** 1 *Lilac Insight Pvt Ltd, Ambience Court, 19*^*th*^* Floor, Unit 1901 & 1902, Sec-19 Vashi, Navi Mumbai 400705 Maharashtra, India.*; 2 *Centre For Human Genetics, Biotech Park, Electronic City, Phase- I, Bangalore-560100, Karnataka, India.*

**Keywords:** Consanguinity, endogamy, inversion, familial translocations, conventional cytogenetics

## Abstract

We present 2 cases of likely rare event. In case 1, 3^rd^ degree consanguineous marriage revealed inv(6) with same break points in parents who were found to be phenotypically normal. The same inv(6) being inherited in progeny but presented with low AMH (anti Mullerian hormone) and high level of FSH (follicular stimulating hormone) leading to polycystic ovarian syndrome/premature ovarian failure. In case 2, a couple was presented with 2^nd^ degree consanguineous marriage and referred for 2 recurrent/ missed abortions. The amounts of shared genes are suggestive of more lethal genetic outcomes and inferred endogamy is a major driver to reproductive fiascoes, the ancestries of which are deeply tied at the meiotic level.

Consanguinity is the distinction of existence to become heir from the same ancestor as another person. The degree of consanguinity can be exemplified with a consanguinity table, where each level of lineal consanguinity (*i.e.*, genera-tion or meiosis) appears as a row, and entities with a collaterally consanguineous relationship share the identical row.

The percentage of consanguinity between any two individuals shows a descend of fourfold as the most current common ancestor recedes one peer group. Consanguinity, as generally implicit, does not depend on the amount of shared DNA within two people's genome. It somewhat sums the number of meioses unscrambling two persons. Due to the effects of pedigree collapse or Ahnenschwund  (loosely decoded: loss of ancestry) or the term implex**,** this does not directly translate into the amount of shared genetic substance. However, different types of consanguineous marriages varies from cross cousin to more detached kindred and their pervasiveness differs by social traditions followed by a community (1, 2). In the Indian context, Hussain et al. analysed the National Family Health Survey (NFHS-1, 1992-1993) and established that the incidence of consanguineous marriage is nearby 12 percent. Nonetheless, it is considerably higher amid Muslims (22 percent)

than other social groups (3).

We restricted our present scope to the health implications in general and exacting in pregnancy outcomes. Earlier literature tells us that, women wedded to their blood kinsfolks experienced a greater amount of fetal wastage and child loss as observed in women married to their distant relatives or non- relatives. Sadaat performed an ecological study using data from 63 countries, thereby raising the hypothesis that offsprings from consanguineous marriages experience higher morbidity and mortality (4). Furthermore, consanguineous unions lead to amplified expression of autosomal recessive disorders (1, 5-7). The progeny of consanguineous unifications may be at increased risk for retreating syndromes because of the manifestation of autosomal recessive gene mutations inherited from a common ancestor. The nearer the biological association between parents, the higher is the probability that their offspring will inherit indistinguishable duplicates of one or more injurious receding genes. For instance, first cousins are expected to share 12.5% (1/8) of their genes. Hence normally, their offspring will be homozygous at 6.25% (1/16) of gene loci (8). In populations of South India, West Asia and North Africa, consanguineous marriages are culturally and socially favored and constitute 20–50% of all marriages. The high consanguinity rates, united by the large family size in some populations, could persuade the manifestation of autosomal recessive ailments, containing very erratic or new syndromes which upsurge the public cognizance of the jeopar-dizes connected with consanguineous marriages. Consanguinity related genetic glitches are sundry; few glaringly observed are multiple congenital disorders, mental retardation, premature ovarian failure, infertility, chromosomal translocations, bad obstetric history, missed abortion and so on. 

## Case Reports

Here we present 2 unusual familial cases which were presented at Lilac Insights cytogenetics department. For both cases, PHA (phytohaemagg-lutinin -M form) stimulated lymphocyte cultures were set up for 72 h in RPMI1640 medium with 10% heat inactivated fetal bovine serum (FBS South American Origin). The stimulated cultures were arrested using Colchicine (10 μg/ml) after 69^th^ hour and fixed cells were obtained after hypotonic treatment using 0.56% KCl (potassium chloride) and three fixative washes. The slides were GTG (Giemsa- trypsin- Giemsa) banded and observed for chromosome analysis using Carl Zeiss, Metasystem software, (Germany).

**Case 1**

**Fig. 1:**
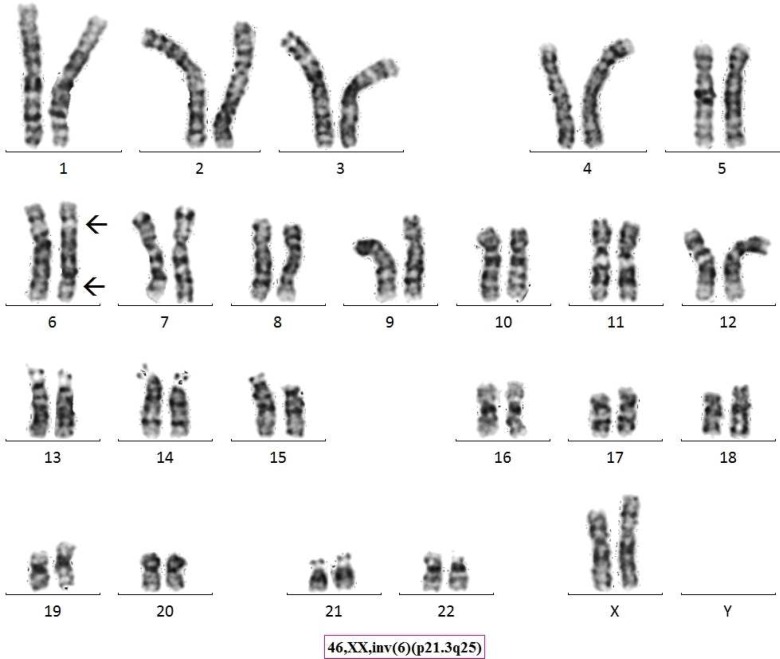
Giemsa-Tyrpsin Banded Karyotype of Proband.

**Fig. 2:**
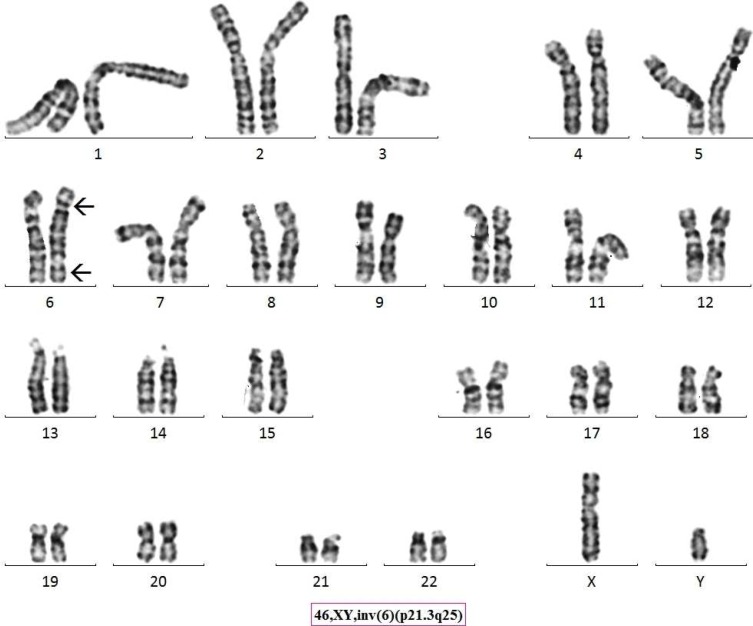
Giemsa-Tyrpsin Banded karyotype image of Proband’s Father

**Fig. 3:**
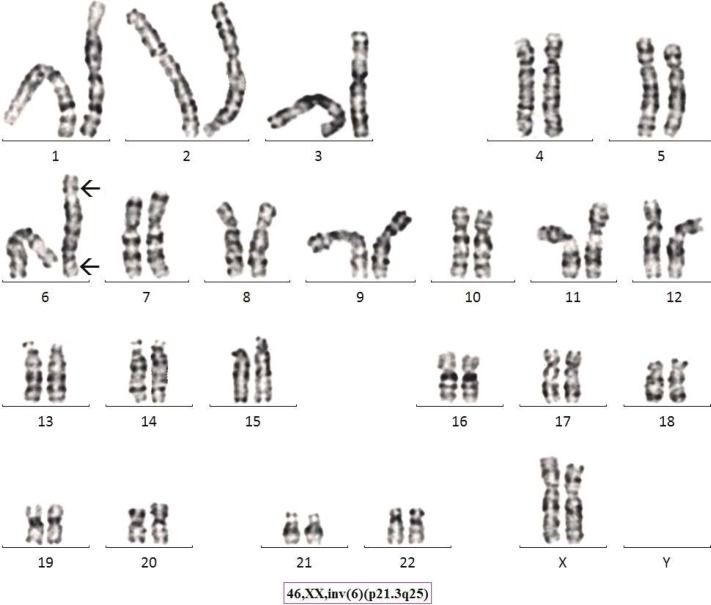
Giemsa-Tyrpsin Banded Karyotype of Proband’s Mother

**Fig. 4:**
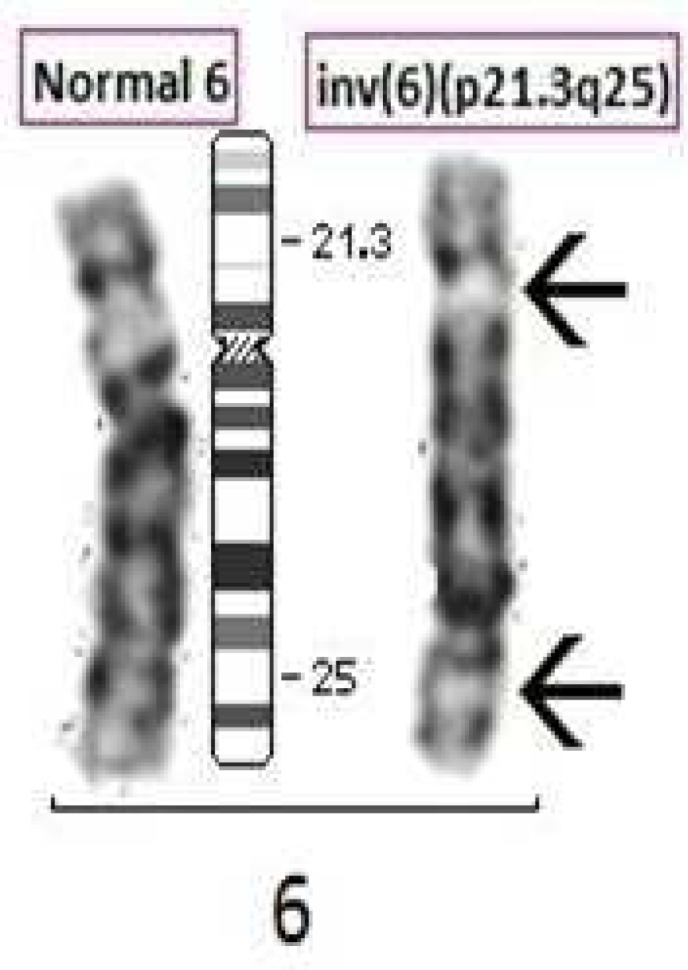
Partial karyotype of Normal Chromosome and Abnormal chromosome 6 with Ideogram of normal Chromosome 6

Chromosome analysis for a 21 year old progeny from 3^rd^ degree consanguineous marriage adult female with menses not attained, revealed a karyotype of 46,XX,inv(6)(p21.3q25) (Fig. 1). Parental karyotype was recommended based on the findings from progeny and it was established that the marriage type was 3^rd^ degree consanguineous. The parents were found to be intelligent, healthy, fertile and normal civil human beings. Parental chromosome analysis revealed exactly similar inversion as it was found in the progeny; 46,XX,inv(6)(p21.3q25) and 46,XY,inv(6)(p21.3q25) (Fig. 2, 3) respectively for mother and father. We found a likely rare event or an unusual case where a 3^rd^ degree consanguineous child inherited the inv(6) from parents, however, the adult child was found to have low AMH (anti Mullerian hormone) and high level of FSH (follicular stimulating hormone) leading to polycystic ovarian syndrome/ premature ovarian failure and did not attain menses at age of 21 years. (Fig. 4 partial karyotype of normal and abnormal chromosome 6).

**Case 2**

A 2^nd^ degree consanguineous marriage couple aged 30 (husband) years and 27 (wife) years respectively were referred for 2 recurrent/ missed abortions. First loss was reported at 1^st^ month after conceiving and second loss was reported at 2^nd^ month after conceiving.

**Fig. 5:**
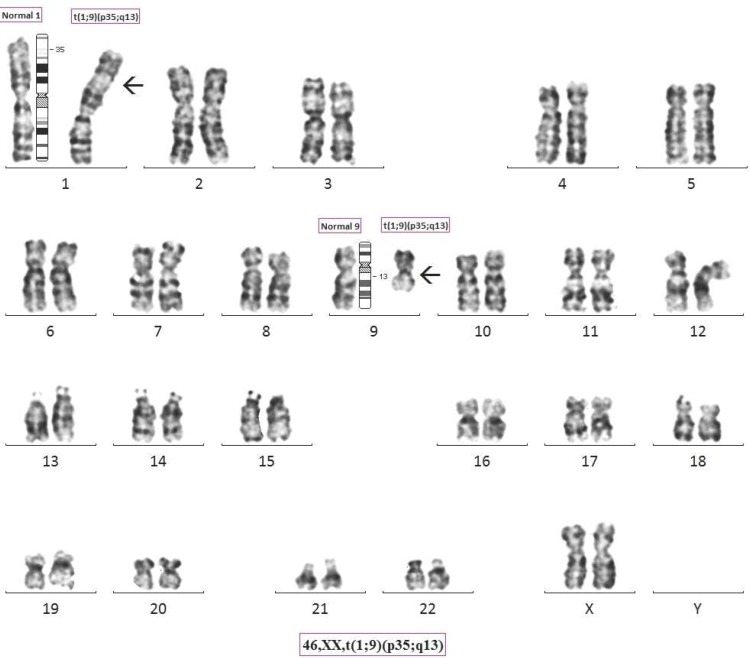
Giemsa Banded Karyotype image of wife

**Fig. 6:**
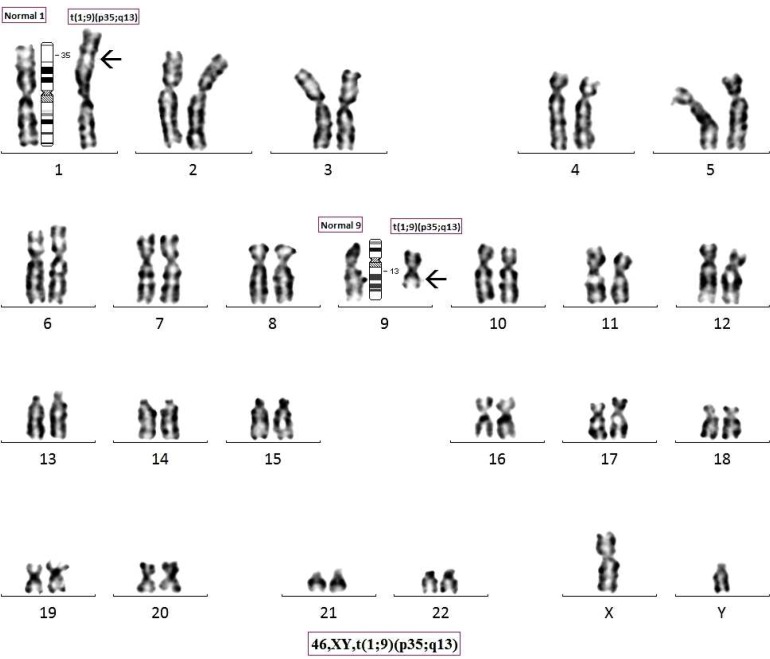
Giemsa Banded Karyotype image of Husband

**Fig. 7:**
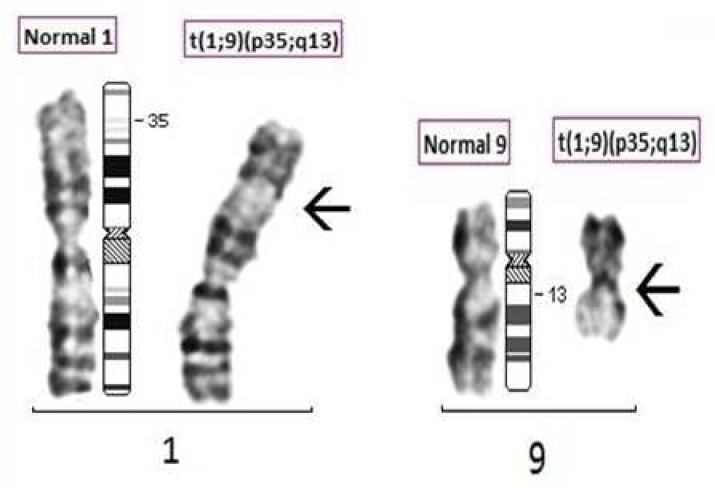
Partial karyotype of normal and abnormal chromosome 1 and 9 and normal chromosome ideogram respectively

Chromosome analysis revealed a karyotype of 46, XX, t(1; 9)(p35; q13) and 46, XY, t(1; 9)(p35; q13) (Fig. 5 and 6) respectively for wife and husband. (Fig. 7 partial karyotypes of normal and abnormal chromosome 1 and 9). Our findings were unusual and are suggestive of 2^nd^ degree consangui-neous marriages leading to and reported with fetal loss and one of the causes of infertility.

## Discussions

**Case 1**

AMH (Anti Mullerian Hormone) is expressed by granulose cells of the ovary during reproductive years and is responsible for limiting the formation of key follicles by barring unwarranted follicular recruitment by FSH (Follicular stimulating hormone). Inversions are poised relocations that seldom cause complications in carriers unless if one of the break points involved has disrupted a gene of importance. Nonetheless, when balanced carriers transmit the rearrangement, it can result in a significant chromosomal imbalance in the offspring. The simple rule is that if the inverted segment is small, because of cross over the deleted and duplicated segments is relatively large, the resultant products are therefore nonviable (9). On the contrary, if there is involvement of large inversion segment, deletion and duplicated segments are small. This development can result in a viable fetus, surviving to term and beyond and is probably seen in our case which may be a rare scientific occurrence and helpful in ongoing research. The frequency of pericentric inversions among the commonest chromosomal rearrang-ements is 1-2%. Pericentric inversions result from a two-break event which occurs between the petite short (p) and the long quartite arms (q) within the chromosome followed by a 180˚ rotation of the intercalary fragment. There is no phenotypic consequence in the majority of pericentric inversion heterozygote carriers with balanced rearrangement as observed in our case. However, miscarriages, infertility can be observed in carriers of a pericen-tric inversion. Transferors of such reorganizations are at jeopardy during meiosis, in order they produ-ce a percentage of abnormal gametes with replica-tion of the region exterior to the inversion segment on one arm of the inverted chromosome and dele-tion of the terminal section on the further arm, and vice versa, it will end up with duplicated/ deficient recombinant chromosomes distal to the breakpoints**.**

**Case 2**

It has been advocated that fertility may be poorer in consanguineous couples due to a failure to initiate pregnancy when the duo share precise HLA haplotypes **(**10), or because of the expression of deleterious genes acting during premature nascent or fetal growth that result in pre- conceptual losses (11). It could be debated that the grander genetic compatibility amongst the mother and developing fetus in a consanguineous pregnancy would lead to abridged rates of involuntary desolation and prenatal losses. Miscarriage or spontaneous abortion is defined as a gestation which ends extemporaneously before the fetus has reached sustainable gestational stage. Thus,the term includes all pregnancy fatalities from the origin until the 24th week of gestation. Recurrent spontaneous abortion (RSA), which occurs in approximately 1-5% of conceptions, is defined as having three or more successive spontaneous miscarriages in the first trimester of gestation. Couples who carry balanced chromosomal rearrangements can produce anomalous gametes with unbalanced chromosomal relocation during gametogenesis and transfer this abnormality to their fetus, which may be the consequence in either RSA or congenital abnormalities. The frequency of uterine abnormalities in women with RSA varies from 2% to 37% and this may play an important role in failure of pregnancy. Previous studies exhibited that the most frequent problem in poised translocation carriers is increased frequency of miscarriage as a result of the formation of unstable gametes. The study also highlighted that carriers of balanced translocation must be followed for life and directed to pre- implantation genetic centers to avoid fetal abnormalities (12). It is suggestive that certain amount of gene sharing occurs among the couple, this adds emphasis why consanguineous marriages lead to higher amount of genetic defects associated with pregnancy wastage, however, to the finest of our knowledge our conclusions in case 1 of inv 6 is, it is a rare event where the parents were normal in terms of fertility, conversely the next generation expressed with fertility problems. Similarly in case 2, consanguinity is of 2^nd^ degree, amount of shared genes are suggestive of more lethal genetic outcomes. Nevertheless, the finding in case 2 is similar to previous study reported in couple with translocation with same breakpoints of chromosome 6 and 16 t(16;6)(p12;q26) was reported in a couple in earlier literature (13) deducing endogamy is a weighty patron to reproductive failures, the roots of which are deeply coupled at the meiotic level. Due to the hereditary transmission of this chromosomal abnormality, cytogenetic investigation for all the siblings and genetic consultation before marriage is highly recommended, this will assuredly lead to a stride to control fetal loss and individuals who are born with inherited genetic defects.

Finally, the excess risk that an autosomal recessive disorder will be expressed in the progeny of a consanguineous union is contrariwise relative to the frequency of the malady allele in the gene pool **(**13). Due to this reason, during the last era many disease inheritable factors that are rare in the general population have been identified and their chromosomal positions mapped by reviewing highly inbred families with multiple affected members. In India, the population is segmented into countless endogamous communities (14) that through time have evolved into distinctive breeding pools. Whether or not a transformation will appear in all communities or be restricted to a single subcaste or segmented community will be reliant on the foundation and the oldness of the community. At least three major migrations into the Indian subcontinent have been acknowledged (14, 15), but across historical time virtually there were smaller passages involving one or several subpopulations. Four basic classes can be defined with respect to the age of mutations. For example, mutations that occurred over 100 generations ago may conceivably be established in all Hindu social group, whereas those of more fresh origin probably have restricted distribution and may be unique to definite subcastes. The smaller the community the greater the probability that founder effect and genetic float will employ a significant effect on the dispersal patterns of specific mutations. Therefore, in the absenteeism of favored consanguineous conjugal, genomic segregation frequently results in an amplified incidence of community- specific inherited diseases. This information is usually unheeded, with the subsequent common hypothesis that where an autosomal recessive syndrome is existing in a clan or community at high incidence consanguinity is certainly implicated.
